# 2-[4-(Piperidin-1-yl)-5*H*-chromeno[2,3-*d*]pyrimidin-2-yl]phenol

**DOI:** 10.1107/S1600536814005625

**Published:** 2014-03-15

**Authors:** Naresh Sharma, Goutam Brahmachari, Suvankar Das, Rajni Kant, Vivek K. Gupta

**Affiliations:** a Post-Graduate Department of Physics & Electronics, University of Jammu, Jammu Tawi 180 006, India; b Laboratory of Natural Products & Organic Synthesis, Department of Chemistry, Visva-Bharati University, Santiniketan 731 235, West Bengal, India

## Abstract

In the title compound, C_22_H_21_N_3_O_2_, the pyrimidine ring is essentially planar [maximum deviation = 0.018 (2) Å] and forms dihedral angles of 22.70 (8) and 0.97 (7)°, respectively, with the fused benzene ring and the hy­droxy-substituted benzene ring. The piperidine ring has a chair conformation and the pyran ring has a flattened twist-boat conformation. The hy­droxy group was refined as disordered over two sets of sites in a 0.702 (4):0.298 (4) ratio. The disorder corresponds to a rotation of approxomiately 180° about the C—C bond connecting the phenol group to the pyrimidine ring and hence, both the major and minor components of disorder form intra­molecular O—H⋯N hydrogen bonds. In the crystal, pairs of weak C—H⋯π inter­actions form inversion dimers. In addition, π–π inter­actions are observed between the pyrimidine ring and the hy­droxy-substituted benzene ring [centroid–centroid separation = 3.739 (2) Å].

## Related literature   

For applications of benzo­pyrano[2,3-*d*]pyrimidines, see: Hadfield *et al.* (1999 ▶); Bruno *et al.* (2001 ▶, 2004 ▶). For general background to benzo­pyrano[2,3-*d*]pyrimidines, see: Brahmachari & Das (2014 ▶). For a related structure, see: Gajera *et al.* (2013 ▶). For standard bond-length data, see: Allen *et al.* (1987 ▶). For conformational analysis, see: Duax & Norton (1975 ▶).
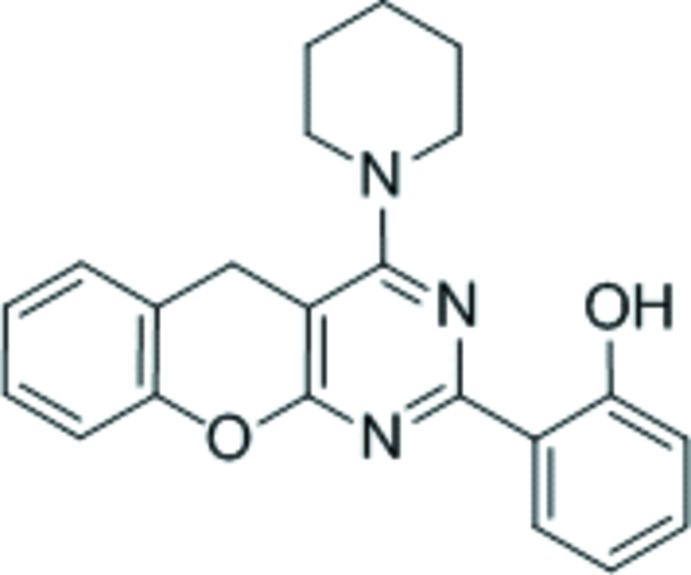


## Experimental   

### 

#### Crystal data  

C_22_H_21_N_3_O_2_
*M* *_r_* = 359.42
Monoclinic, 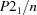

*a* = 9.9826 (5) Å
*b* = 15.8773 (7) Å
*c* = 12.2197 (6) Å
β = 109.381 (6)°
*V* = 1827.03 (15) Å^3^
*Z* = 4
Mo *K*α radiation
μ = 0.09 mm^−1^
*T* = 293 K
0.30 × 0.20 × 0.20 mm

#### Data collection  

Oxford Diffraction Xcalibur Sapphire3 diffractometer
Absorption correction: multi-scan (*CrysAlis RED*; Oxford Diffraction, 2010 ▶) *T*_min_ = 0.975, *T*_max_ = 0.983
13349 measured reflections
3576 independent reflections
1918 reflections with *I* > 2σ(*I*)
*R*_int_ = 0.048

#### Refinement  

*R*[*F* ^2^ > 2σ(*F* ^2^)] = 0.060
*wR*(*F* ^2^) = 0.133
*S* = 1.03
3576 reflections
254 parameters
12 restraints
H-atom parameters constrained
Δρ_max_ = 0.13 e Å^−3^
Δρ_min_ = −0.16 e Å^−3^

### 

Data collection: *CrysAlis PRO* (Oxford Diffraction, 2010 ▶); cell refinement: *CrysAlis PRO*; data reduction: *CrysAlis PRO*; program(s) used to solve structure: *SHELXS97* (Sheldrick, 2008 ▶); program(s) used to refine structure: *SHELXL97* (Sheldrick, 2008 ▶); molecular graphics: *ORTEP-3 for Windows* (Farrugia, 2012 ▶) and *PLATON* (Spek, 2009 ▶); software used to prepare material for publication: *PLATON*.

## Supplementary Material

Crystal structure: contains datablock(s) I, New_Global_Publ_Block. DOI: 10.1107/S1600536814005625/lh5695sup1.cif

Structure factors: contains datablock(s) I. DOI: 10.1107/S1600536814005625/lh5695Isup2.hkl

Supporting information file. DOI: 10.1107/S1600536814005625/lh5695Isup3.cml

CCDC reference: 975917

Additional supporting information:  crystallographic information; 3D view; checkCIF report

## Figures and Tables

**Table 1:** Hydrogen-bond geometry (Å, °) *Cg* is the centroid of the C6–C9/C12/C13 ring.

*D*—H⋯*A*	*D*—H	H⋯*A*	*D*⋯*A*	*D*—H⋯*A*
O27*A*—H27*A*⋯N3	0.82	1.78	2.535 (3)	151
O27*B*—H27*B*⋯N1	0.82	1.83	2.551 (3)	146
C5—H5*B*⋯*Cg* ^i^	0.97	2.67	3.59	159

Symmetry code: (i) 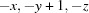.

**Fig. 1.**
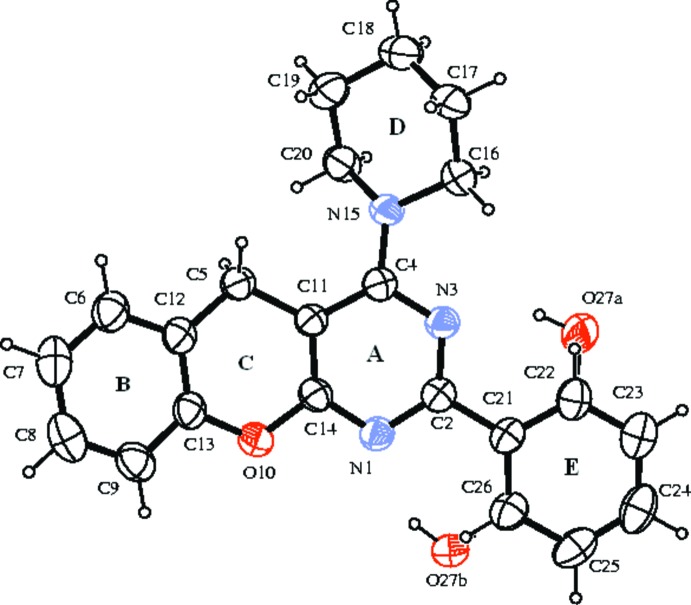
The molecular structure of (I) with ellipsoids drawn at the 40% probability level. H atoms are shown as small spheres of arbitrary radii. Both disorder components are shown.

**Fig. 2.**
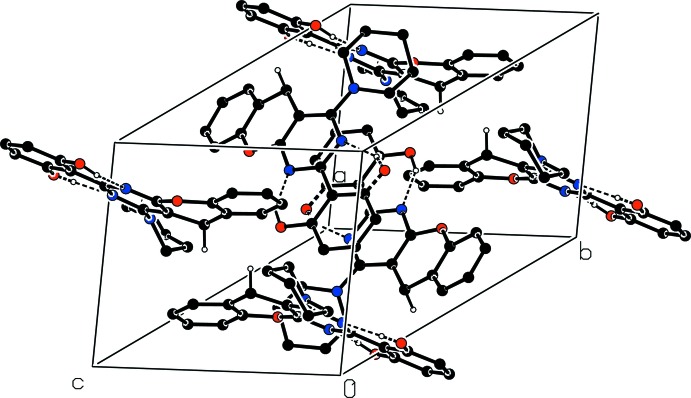
Part of the crystal structure with hydrogen bonds shown as dashed lines. Only the H atoms involved in hydrogen bonds and weak C—H···π interactions are shown.

## supplementary crystallographic information

### 1. Comment

Benzopyrano[2,3-*d*]pyrimidines have gained much attention as significant medicinal scaffolds due to their inherent and multidirectional pharmaceutical potentials that include anti-inflammatory, analgesic, and anti-aggregating activities (Bruno *et al.*, 2001,2004). More importantly, such chemical entities have been found to exhibit potent *in vivo* antitumor as well as *in vitro* cytotoxic activity against various cancer cell lines causing considerable degree of perturbation in cell cycle kinetics (Hadfield *et al.*, 1999). Very recently, we have developed a straight forward and efficient pseudo four-component one-pot synthesis of diverse benzopyrano[2,3-*d*]pyrimidine scaffolds in good yields using commercially available sodium formate as an inexpensive and non-toxic catalyst (Brahmachari & Das, 2014). Herein, we wish to report the environmentally benign one-pot synthesis and X-ray crystal structure of 2-(4-(piperidin-1-yl)-5*H*-chromeno[2,3-*d*]pyrimidin-2-yl)phenol.

The molecular structure of the title compound (I) is shown in Fig. 1. In (I), the expected values for the bond-lengths are observed (Allen *et al.*, 1987) and the distances are comparable to a closely related structure (Gajera *et al.*, 2013). The pyrimidine ring (A) is essentially planar with a maximum deviation of 0.019 (2) Å for C4. This ring forms dihedral angles of 22.70 (8)° and 0.97 (7)°, respectively, with the fused benzene ring (B) and hydroxy-substituted benzene ring (E). The pyran ring (C) adopts a flattened twist-boat conformation with asymmetry parameters [ΔCs(C5—C10)=2.61, ΔC2(C11—C14)=2.66] and the piperidine ring (D) adopts chair conformation with asymmetry parameters [ΔCs(C17—C20)=2.67, ΔC2(C17—C8)=0.2] (Duax & Norton, 1975). The hydroxy group was refined as disordered over two sets of sites in a 0.702 (4): 0.298 (4) ratio. The disorder corresponds to a rotation of approxomiately 180° about the C2—C21 bond and hence, both the major and minor components of disorder form intramolecular O—H···N hydrogen bonds (see Table 1). In the crystal, pairs of weak C—H···π interactions form inversion dimers. In addition, π–π interactions are observed between the pyrimidine ring and hydroxy-substituted benzene ring [centroid–centroid seperation = 3.739 (2) Å, interplanar spacing = 3.534 Å, centriod shift = 1.22 Å, symmetry code: 1 - *x*,1 - *y*,1 - *z*]. The crystal packing is shown in Fig. 2.

### 2. Experimental

Infrared spectra were recorded using a Shimadzu (FT—IR 8400S) FT—IR spectrophotometer using KBr disc. 1H and 13 C NMR spectra was obtained at 400 and 100 MHz, respectively, using Bruker DRX spectrometer and CDCl_3_ and DMSO-d6 as solvents. Elemental analysis was performed with an Elementar Vario EL III Carlo Erba 1108 micro-analyzer instrument. Melting point was recorded on a Sunvic melting point apparatus and is uncorrected. TLC was performed using silica gel 60 F254 (Merck) plates. An oven-dried screw cap test tube was charged with a magnetic stir bar, salicylaldehyde (2 mmol), malononitrile (1 mmol), piperidine (1 mmol), and sodium formate (10 mol%) in 4 ml e thanol. The reaction mixture was stirred at room temperature for 12 h. On completion of the reaction as monitored by TLC, the product was precipitated out and filtered; the filtrate was preserved for reuse. The crude residue was washed with water followed by ethanol to obtain pure product 1, characterized by elemental analyses as well as spectral studies including FT—IR, 1*H*-NMR and 13 C-NMR. The title compound (50 mg) was dissolved in 10 ml DMSO and left for several days at ambient temperature which yielded single crystals suitable for X-ray diffraction.

### 3. Refinement

All H atoms were geometrically fixed and allowed to ride on their parent atoms, with C—H distances of 0.93–0.97 Å, O—H = 0.82Å and with *U*_iso_(H) = 1.2*U*_eq_(C) or *U*_iso_(H) = 1.5*U*_eq_(O).

### Crystal data

**Table:**

C_22_H_21_N_3_O_2_	*F*(000) = 760
*M**_r_* = 359.42	*D*_x_ = 1.307 Mg m^−^^3^
Monoclinic, *P*2_1_/*n*	Mo *K*α radiation, λ = 0.71073 Å
Hall symbol: -P 2yn	Cell parameters from 4022 reflections
*a* = 9.9826 (5) Å	θ = 3.4–29.1°
*b* = 15.8773 (7) Å	µ = 0.09 mm^−^^1^
*c* = 12.2197 (6) Å	*T* = 293 K
β = 109.381 (6)°	Block, colourless
*V* = 1827.03 (15) Å^3^	0.30 × 0.20 × 0.20 mm
*Z* = 4	

### Data collection

**Table:**

Oxford Diffraction Xcalibur Sapphire3 diffractometer	3576 independent reflections
Radiation source: fine-focus sealed tube	1918 reflections with *I* > 2σ(*I*)
Graphite monochromator	*R*_int_ = 0.048
Detector resolution: 16.1049 pixels mm^-1^	θ_max_ = 26.0°, θ_min_ = 3.4°
ω scans	*h* = −12→10
Absorption correction: multi-scan (*CrysAlis RED*; Oxford Diffraction, 2010)	*k* = −19→19
*T*_min_ = 0.975, *T*_max_ = 0.983	*l* = −15→15
13349 measured reflections	

### Refinement

**Table:**

Refinement on *F*^2^	Primary atom site location: structure-invariant direct methods
Least-squares matrix: full	Secondary atom site location: difference Fourier map
*R*[*F*^2^ > 2σ(*F*^2^)] = 0.060	Hydrogen site location: inferred from neighbouring sites
*wR*(*F*^2^) = 0.133	H-atom parameters constrained
*S* = 1.03	*w* = 1/[σ^2^(*F*_o_^2^) + (0.0481*P*)^2^ + 0.0647*P*] where *P* = (*F*_o_^2^ + 2*F*_c_^2^)/3
3576 reflections	(Δ/σ)_max_ < 0.001
254 parameters	Δρ_max_ = 0.13 e Å^−^^3^
12 restraints	Δρ_min_ = −0.16 e Å^−^^3^

### Special details

**Table:**

Experimental. *CrysAlis PRO*, Agilent Technologies, Version 1.171.36.28 (release 01–02-2013 CrysAlis171. NET) (compiled Feb 1 2013,16:14:44) Empirical absorption correction using spherical harmonics, implemented in SCALE3 ABSPACK scaling algorithm.
Geometry. All e.s.d.'s (except the e.s.d. in the dihedral angle between two l.s. planes) are estimated using the full covariance matrix. The cell e.s.d.'s are taken into account individually in the estimation of e.s.d.'s in distances, angles and torsion angles; correlations between e.s.d.'s in cell parameters are only used when they are defined by crystal symmetry. An approximate (isotropic) treatment of cell e.s.d.'s is used for estimating e.s.d.'s involving l.s. planes.
Refinement. Refinement of *F*^2^ against ALL reflections. The weighted *R*-factor *wR* and goodness of fit *S* are based on *F*^2^, conventional *R*-factors *R* are based on *F*, with *F* set to zero for negative *F*^2^. The threshold expression of *F*^2^ > σ(*F*^2^) is used only for calculating *R*-factors(gt) *etc*. and is not relevant to the choice of reflections for refinement. *R*-factors based on *F*^2^ are statistically about twice as large as those based on *F*, and *R*- factors based on ALL data will be even larger.

### Fractional atomic coordinates and isotropic or equivalent isotropic displacement parameters (Å^2^)

**Table:**

	*x*	*y*	*z*	*U*_iso_*/*U*_eq_	Occ. (<1)
C2	0.1453 (2)	0.07319 (14)	0.0435 (2)	0.0477 (6)	
C4	0.3129 (2)	−0.03037 (14)	0.1148 (2)	0.0463 (6)	
C5	0.3928 (2)	−0.06426 (14)	0.33523 (19)	0.0507 (6)	
H5A	0.3894	−0.1235	0.3152	0.061*	
H5B	0.4916	−0.0469	0.3622	0.061*	
C6	0.3630 (3)	−0.10672 (16)	0.5247 (2)	0.0647 (8)	
H6	0.4218	−0.1527	0.5280	0.078*	
C7	0.3085 (3)	−0.09410 (19)	0.6134 (2)	0.0742 (9)	
H7	0.3312	−0.1312	0.6758	0.089*	
C8	0.2209 (3)	−0.0268 (2)	0.6095 (3)	0.0756 (9)	
H8	0.1837	−0.0185	0.6691	0.091*	
C9	0.1882 (3)	0.02815 (18)	0.5177 (2)	0.0658 (8)	
H9	0.1296	0.0742	0.5147	0.079*	
C11	0.3132 (2)	−0.01433 (14)	0.2289 (2)	0.0443 (6)	
C12	0.3321 (3)	−0.05240 (15)	0.4307 (2)	0.0499 (6)	
C13	0.2437 (3)	0.01392 (16)	0.4299 (2)	0.0522 (7)	
C14	0.2228 (3)	0.04870 (15)	0.2367 (2)	0.0483 (6)	
C16	0.3602 (3)	−0.11949 (17)	−0.0314 (2)	0.0693 (8)	
H16A	0.4132	−0.0854	−0.0688	0.083*	
H16B	0.2598	−0.1115	−0.0730	0.083*	
C17	0.3982 (3)	−0.21068 (17)	−0.0359 (2)	0.0731 (9)	
H17A	0.3828	−0.2264	−0.1159	0.088*	
H17B	0.3360	−0.2449	−0.0078	0.088*	
C18	0.5505 (3)	−0.22899 (19)	0.0357 (3)	0.0843 (10)	
H18A	0.6137	−0.2019	0.0009	0.101*	
H18B	0.5674	−0.2892	0.0374	0.101*	
C19	0.5809 (3)	−0.19662 (19)	0.1583 (2)	0.0783 (9)	
H19A	0.5263	−0.2288	0.1963	0.094*	
H19B	0.6808	−0.2040	0.2020	0.094*	
C20	0.5425 (3)	−0.10490 (16)	0.1567 (2)	0.0618 (7)	
H20A	0.5606	−0.0853	0.2355	0.074*	
H20B	0.6010	−0.0723	0.1229	0.074*	
C21	0.0531 (2)	0.12112 (13)	−0.0572 (2)	0.0488 (6)	
C22	0.0519 (3)	0.10222 (18)	−0.1690 (2)	0.0599 (7)	
H22	0.1079	0.0582	−0.1794	0.072*	0.702 (4)
O27A	0.1226 (5)	0.0461 (3)	−0.1909 (5)	0.076 (3)	0.298 (4)
H27A	0.1672	0.0216	−0.1308	0.114*	0.298 (4)
C23	−0.0300 (3)	0.14697 (19)	−0.2643 (3)	0.0711 (8)	
H23	−0.0277	0.1340	−0.3379	0.085*	
C24	−0.1140 (3)	0.2101 (2)	−0.2497 (3)	0.0745 (9)	
H24	−0.1694	0.2404	−0.3140	0.089*	
C25	−0.1190 (3)	0.23027 (16)	−0.1420 (3)	0.0728 (9)	
H25	−0.1787	0.2729	−0.1336	0.087*	
C26	−0.0337 (3)	0.18620 (15)	−0.0448 (3)	0.0604 (7)	
H26	−0.0350	0.2006	0.0286	0.072*	0.298 (4)
O27B	−0.0379 (3)	0.21092 (14)	0.0561 (2)	0.0703 (11)	0.702 (4)
H27B	0.0250	0.1872	0.1079	0.105*	0.702 (4)
N1	0.1407 (2)	0.09492 (12)	0.14824 (18)	0.0522 (5)	
N3	0.2261 (2)	0.01277 (12)	0.02367 (17)	0.0499 (5)	
N15	0.3930 (2)	−0.09248 (12)	0.08946 (17)	0.0536 (6)	
O10	0.20328 (18)	0.07135 (10)	0.33851 (15)	0.0621 (5)	

### Atomic displacement parameters (Å^2^)

**Table:**

	*U*^11^	*U*^22^	*U*^33^	*U*^12^	*U*^13^	*U*^23^
C2	0.0481 (15)	0.0410 (14)	0.0520 (16)	−0.0057 (12)	0.0137 (12)	−0.0011 (12)
C4	0.0432 (14)	0.0424 (14)	0.0499 (15)	−0.0060 (12)	0.0107 (12)	−0.0062 (12)
C5	0.0486 (15)	0.0474 (15)	0.0501 (16)	0.0009 (12)	0.0083 (12)	−0.0074 (12)
C6	0.0749 (19)	0.0566 (18)	0.0548 (18)	−0.0014 (14)	0.0110 (15)	−0.0021 (15)
C7	0.095 (2)	0.071 (2)	0.0508 (18)	−0.0174 (19)	0.0165 (16)	0.0041 (15)
C8	0.082 (2)	0.093 (2)	0.055 (2)	−0.0225 (19)	0.0271 (17)	−0.0147 (18)
C9	0.0647 (19)	0.073 (2)	0.0583 (19)	−0.0039 (15)	0.0189 (15)	−0.0157 (16)
C11	0.0454 (14)	0.0373 (13)	0.0461 (15)	−0.0035 (11)	0.0099 (12)	−0.0056 (11)
C12	0.0515 (15)	0.0461 (15)	0.0446 (15)	−0.0074 (13)	0.0061 (12)	−0.0082 (12)
C13	0.0559 (16)	0.0523 (16)	0.0424 (15)	−0.0050 (13)	0.0082 (13)	−0.0069 (13)
C14	0.0527 (15)	0.0434 (14)	0.0469 (15)	−0.0046 (12)	0.0139 (12)	−0.0095 (12)
C16	0.080 (2)	0.0724 (19)	0.0500 (17)	0.0152 (16)	0.0138 (14)	−0.0087 (14)
C17	0.084 (2)	0.0658 (19)	0.0637 (19)	0.0169 (16)	0.0171 (16)	−0.0174 (15)
C18	0.085 (2)	0.080 (2)	0.081 (2)	0.0266 (18)	0.0185 (18)	−0.0180 (17)
C19	0.0648 (19)	0.087 (2)	0.072 (2)	0.0243 (17)	0.0071 (15)	−0.0029 (17)
C20	0.0479 (16)	0.075 (2)	0.0596 (17)	−0.0004 (14)	0.0144 (13)	−0.0148 (14)
C21	0.0495 (15)	0.0381 (14)	0.0547 (16)	−0.0058 (12)	0.0117 (12)	0.0047 (12)
C22	0.0549 (17)	0.0660 (19)	0.0544 (18)	−0.0102 (15)	0.0121 (14)	0.0054 (15)
O27A	0.057 (4)	0.109 (6)	0.061 (4)	0.019 (4)	0.017 (3)	0.009 (4)
C23	0.0653 (19)	0.079 (2)	0.0622 (19)	−0.0111 (17)	0.0115 (15)	0.0125 (17)
C24	0.070 (2)	0.070 (2)	0.069 (2)	−0.0155 (17)	0.0042 (16)	0.0223 (17)
C25	0.0633 (19)	0.0512 (18)	0.092 (2)	0.0057 (14)	0.0092 (17)	0.0127 (17)
C26	0.0659 (18)	0.0452 (16)	0.0648 (19)	−0.0019 (14)	0.0146 (15)	0.0029 (14)
O27B	0.083 (2)	0.0662 (18)	0.061 (2)	0.0287 (14)	0.0220 (14)	0.0006 (14)
N1	0.0570 (13)	0.0442 (12)	0.0518 (13)	0.0029 (10)	0.0132 (10)	−0.0018 (10)
N3	0.0503 (12)	0.0461 (12)	0.0507 (13)	0.0030 (10)	0.0132 (10)	−0.0030 (10)
N15	0.0529 (13)	0.0540 (13)	0.0486 (13)	0.0093 (10)	0.0097 (10)	−0.0106 (10)
O10	0.0786 (13)	0.0545 (11)	0.0527 (11)	0.0138 (9)	0.0208 (9)	−0.0054 (9)

### Geometric parameters (Å, º)

**Table:**

C2—N3	1.326 (3)	C17—C18	1.509 (4)
C2—N1	1.341 (3)	C17—H17A	0.9700
C2—C21	1.479 (3)	C17—H17B	0.9700
C4—N3	1.349 (3)	C18—C19	1.517 (3)
C4—N15	1.368 (3)	C18—H18A	0.9700
C4—C11	1.416 (3)	C18—H18B	0.9700
C5—C12	1.495 (3)	C19—C20	1.504 (3)
C5—C11	1.504 (3)	C19—H19A	0.9700
C5—H5A	0.9700	C19—H19B	0.9700
C5—H5B	0.9700	C20—N15	1.458 (3)
C6—C7	1.379 (4)	C20—H20A	0.9700
C6—C12	1.387 (3)	C20—H20B	0.9700
C6—H6	0.9300	C21—C26	1.388 (3)
C7—C8	1.371 (4)	C21—C22	1.395 (3)
C7—H7	0.9300	C22—O27A	1.220 (5)
C8—C9	1.373 (4)	C22—C23	1.378 (3)
C8—H8	0.9300	C22—H22	0.9300
C9—C13	1.379 (3)	O27A—H27A	0.8200
C9—H9	0.9300	C23—C24	1.357 (4)
C11—C14	1.372 (3)	C23—H23	0.9300
C12—C13	1.371 (3)	C24—C25	1.371 (4)
C13—O10	1.394 (3)	C24—H24	0.9300
C14—N1	1.338 (3)	C25—C26	1.397 (4)
C14—O10	1.370 (3)	C25—H25	0.9300
C16—N15	1.467 (3)	C26—O27B	1.309 (3)
C16—C17	1.503 (3)	C26—H26	0.9300
C16—H16A	0.9700	O27B—H27B	0.8200
C16—H16B	0.9700		
			
N3—C2—N1	125.1 (2)	C17—C18—C19	109.9 (2)
N3—C2—C21	118.0 (2)	C17—C18—H18A	109.7
N1—C2—C21	116.9 (2)	C19—C18—H18A	109.7
N3—C4—N15	116.3 (2)	C17—C18—H18B	109.7
N3—C4—C11	120.8 (2)	C19—C18—H18B	109.7
N15—C4—C11	122.8 (2)	H18A—C18—H18B	108.2
C12—C5—C11	111.9 (2)	C20—C19—C18	110.4 (2)
C12—C5—H5A	109.2	C20—C19—H19A	109.6
C11—C5—H5A	109.2	C18—C19—H19A	109.6
C12—C5—H5B	109.2	C20—C19—H19B	109.6
C11—C5—H5B	109.2	C18—C19—H19B	109.6
H5A—C5—H5B	107.9	H19A—C19—H19B	108.1
C7—C6—C12	121.5 (3)	N15—C20—C19	110.3 (2)
C7—C6—H6	119.2	N15—C20—H20A	109.6
C12—C6—H6	119.2	C19—C20—H20A	109.6
C8—C7—C6	120.0 (3)	N15—C20—H20B	109.6
C8—C7—H7	120.0	C19—C20—H20B	109.6
C6—C7—H7	120.0	H20A—C20—H20B	108.1
C7—C8—C9	120.0 (3)	C26—C21—C22	117.6 (2)
C7—C8—H8	120.0	C26—C21—C2	122.1 (2)
C9—C8—H8	120.0	C22—C21—C2	120.3 (2)
C8—C9—C13	118.9 (3)	O27A—C22—C23	114.5 (4)
C8—C9—H9	120.5	O27A—C22—C21	123.7 (4)
C13—C9—H9	120.5	C23—C22—C21	121.8 (3)
C14—C11—C4	114.4 (2)	C23—C22—H22	119.1
C14—C11—C5	119.7 (2)	C21—C22—H22	119.1
C4—C11—C5	125.7 (2)	C22—O27A—H27A	109.5
C13—C12—C6	116.7 (2)	C24—C23—C22	119.3 (3)
C13—C12—C5	121.2 (2)	C24—C23—H23	120.4
C6—C12—C5	122.1 (2)	C22—C23—H23	120.4
C12—C13—C9	122.9 (3)	C23—C24—C25	121.3 (3)
C12—C13—O10	121.5 (2)	C23—C24—H24	119.3
C9—C13—O10	115.6 (2)	C25—C24—H24	119.3
N1—C14—O10	110.9 (2)	C24—C25—C26	119.5 (3)
N1—C14—C11	125.8 (2)	C24—C25—H25	120.2
O10—C14—C11	123.3 (2)	C26—C25—H25	120.2
N15—C16—C17	110.1 (2)	O27B—C26—C21	122.8 (3)
N15—C16—H16A	109.6	O27B—C26—C25	116.7 (3)
C17—C16—H16A	109.6	C21—C26—C25	120.5 (3)
N15—C16—H16B	109.6	C21—C26—H26	119.8
C17—C16—H16B	109.6	C25—C26—H26	119.8
H16A—C16—H16B	108.2	C26—O27B—H27B	109.5
C16—C17—C18	112.5 (2)	C14—N1—C2	115.1 (2)
C16—C17—H17A	109.1	C2—N3—C4	118.7 (2)
C18—C17—H17A	109.1	C4—N15—C20	122.37 (19)
C16—C17—H17B	109.1	C4—N15—C16	119.1 (2)
C18—C17—H17B	109.1	C20—N15—C16	111.89 (19)
H17A—C17—H17B	107.8	C14—O10—C13	117.75 (19)
			
C12—C6—C7—C8	0.4 (4)	C26—C21—C22—C23	1.1 (2)
C6—C7—C8—C9	−0.4 (4)	C2—C21—C22—C23	−178.3 (2)
C7—C8—C9—C13	0.6 (4)	O27A—C22—C23—C24	179.2 (2)
N3—C4—C11—C14	−1.7 (3)	C21—C22—C23—C24	−1.3 (3)
N15—C4—C11—C14	−178.1 (2)	C22—C23—C24—C25	0.0 (4)
N3—C4—C11—C5	173.0 (2)	C23—C24—C25—C26	1.4 (4)
N15—C4—C11—C5	−3.4 (4)	C22—C21—C26—O27B	−177.9 (2)
C12—C5—C11—C14	15.2 (3)	C2—C21—C26—O27B	1.5 (3)
C12—C5—C11—C4	−159.2 (2)	C22—C21—C26—C25	0.3 (3)
C7—C6—C12—C13	−0.6 (4)	C2—C21—C26—C25	179.8 (2)
C7—C6—C12—C5	178.7 (2)	C24—C25—C26—O27B	176.8 (2)
C11—C5—C12—C13	−17.3 (3)	C24—C25—C26—C21	−1.5 (4)
C11—C5—C12—C6	163.5 (2)	O10—C14—N1—C2	−175.57 (18)
C6—C12—C13—C9	0.8 (4)	C11—C14—N1—C2	3.3 (3)
C5—C12—C13—C9	−178.4 (2)	N3—C2—N1—C14	−2.0 (3)
C6—C12—C13—O10	−178.6 (2)	C21—C2—N1—C14	178.36 (19)
C5—C12—C13—O10	2.1 (3)	N1—C2—N3—C4	−0.9 (3)
C8—C9—C13—C12	−0.8 (4)	C21—C2—N3—C4	178.68 (18)
C8—C9—C13—O10	178.7 (2)	N15—C4—N3—C2	179.44 (19)
C4—C11—C14—N1	−1.5 (3)	C11—C4—N3—C2	2.9 (3)
C5—C11—C14—N1	−176.5 (2)	N3—C4—N15—C20	137.0 (2)
C4—C11—C14—O10	177.2 (2)	C11—C4—N15—C20	−46.5 (3)
C5—C11—C14—O10	2.2 (3)	N3—C4—N15—C16	−12.1 (3)
N15—C16—C17—C18	−54.4 (3)	C11—C4—N15—C16	164.4 (2)
C16—C17—C18—C19	53.0 (3)	C19—C20—N15—C4	148.4 (2)
C17—C18—C19—C20	−54.2 (3)	C19—C20—N15—C16	−60.5 (3)
C18—C19—C20—N15	58.2 (3)	C17—C16—N15—C4	−149.8 (2)
N3—C2—C21—C26	−179.10 (19)	C17—C16—N15—C20	58.0 (3)
N1—C2—C21—C26	0.6 (3)	N1—C14—O10—C13	160.0 (2)
N3—C2—C21—C22	0.4 (3)	C11—C14—O10—C13	−18.9 (3)
N1—C2—C21—C22	−179.98 (17)	C12—C13—O10—C14	16.6 (3)
C26—C21—C22—O27A	−179.45 (14)	C9—C13—O10—C14	−162.9 (2)
C2—C21—C22—O27A	1.1 (2)		

### Hydrogen-bond geometry (Å, º)

Cg is the centroid of the C6–C9/C12/C13 ring.

**Table:**

*D*—H···*A*	*D*—H	H···*A*	*D*···*A*	*D*—H···*A*
O27*A*—H27*A*···N3	0.82	1.78	2.535 (3)	151
O27*B*—H27*B*···N1	0.82	1.83	2.551 (3)	146
C5—H5*B*···*Cg*^i^	0.97	2.67	3.59	159

Symmetry code: (i) −*x*, −*y*+1, −*z*.
